# Bone marrow mesenchymal stem cells enhance angiogenesis and promote fat retention in fat grafting via polarized macrophages

**DOI:** 10.1186/s13287-022-02709-2

**Published:** 2022-02-04

**Authors:** Juanli Dang, Jizhong Yang, Zhou Yu, Lin Chen, Zhaoxiang Zhang, Kai Wang, Jiezhang Tang, Chenggang Yi

**Affiliations:** 1 Department of Plastic and Reconstructive Surgery, Xijing Hospital, Fourth Military Medical University, Xi’an, China; 2 Department of Plastic Surgery, The Second Affiliated Hospital, Medical School, Zhejiang University, Hangzhou, China

**Keywords:** Bone marrow mesenchymal stem cells, Fat grafting, Macrophage polarization, Angiogenesis

## Abstract

**Background:**

Fat grafting is one of the most common soft tissue filling methods in plastic surgery. Bone marrow mesenchymal stem cell (BM-MSC) transplantation is an effective method for improving graft retention. However, the role of BM-MSCs in fat transplantation is not completely clear.

**Methods:**

Human fat particles, together with BM-MSCs or PBS as a control, were subcutaneously transplanted into the backs of nude mice. Samples were taken on days 14, 30 and 90 post-grafting to calculate the fat graft retention rate, and tissue staining was evaluated. Furthermore, macrophages were treated with BM-MSC conditioned medium (BM-MSC-CM) to identify the beneficial component secreted by these stem cells.

**Results:**

In this study, we found that BM-MSCs improved retention by enhancing angiogenesis in fat grafting. Further analysis revealed that BM-MSCs could significantly inhibit the expression of the proinflammatory M1 macrophage markers interleukin (IL)-1β, tumor necrosis factor-α (TNF-α) and IL-6 in the early stages of fat grafting and promote the expression of the anti-inflammatory M2 macrophage markers Arg1, IL-10 and VEGF. Furthermore, our results showed that IL-10 secreted by BM-MSCs induced M2 macrophage polarization in vitro.

**Conclusions:**

BM-MSC transplantation can improve the fat retention rate and promote angiogenesis, which may be related to M2 macrophages. These results help elucidate the role of BM-MSCs in fat grafting.

## Introduction

Fat grafting is increasingly being used in the treatment of soft tissue defects and reconstructive surgery [1, 2]. A major limitation is the high absorption rate, which leads to instability in clinical application [3]. Early rapid neovascularization plays a key role among the numerous factors that can improve the retention of transferred fat [4]. Mesenchymal stem cells (MSCs) are multifunctional stem cells that have been explored in the applied fields of orthopaedic regeneration, pain treatment, arthritis, asthma and so on [5–7]. In our previous study, we found that bone marrow mesenchymal stem cells (BM-MSCs) isolated from rabbits could improve fat retention and enhance angiogenesis [8]. However, the mechanisms underlying the improvement in the retention rate and enhancement of angiogenesis in the context of fat grafting are unclear.

Macrophages are important innate immune cells in the human body and play an important role in fat tissue biology [9]. Moreover, macrophages can be divided into two phenotypes: “classically activated” M1 macrophages and “alternatively activated” M2 macrophages [10]. M1 macrophages exert proinflammatory activity by producing proinflammatory cytokines and proteolytic enzymes, while M2 macrophages have anti-inflammatory/regenerative abilities, demonstrated by the secretion of interleukin (IL)-10 or TGF-β [11]. These cytokines help to resolve inflammation and facilitate tissue remodelling. M2 macrophages secrete and release many angiogenic factors (chemokines, cytokines, and growth factors) to stimulate endothelial cell migration and proliferation [12]. Studies have shown that the addition of M2 macrophages can enhance angiogenesis in fat grafts [13]. Thus, increasing the number or proportion of M2 macrophages in the recipient area is an attractive treatment strategy to increase the retention rate of fat grafts. However, it is difficult to enrich M2 macrophages for clinical application. Therefore, researchers have begun to seek alternative strategies to regulate macrophage polarization.

MSCs are the most widely used stem cells. Compared with other types of stem cells, BM-MSCs have significant immunomodulatory abilities [14]. The interaction between immune cells and stem cells in tissue regeneration has been known for a long time. When the tissue barrier is destroyed, stem cells recruit immune cells to the wound site, which may create a cascade and amplify the regenerative response. BM-MSCs can reduce inflammation and inflammatory responses and promote tissue regeneration by inducing macrophage repolarization from the M1 phenotype to the M2 phenotype in various disease models [15]. However, it is unknown whether BM-MSCs exert their therapeutic effects by modulating the macrophage phenotype in the context of fat grafting.

There is evidence that MSCs have extensive therapeutic potential and show some promise in the context of fat grafting [8], but the underlying mechanisms remain unclear. Clarifying the related mechanisms will provide ideas for easy and reliable treatment methods to improve fat grafting. In this study, we confirmed that BM-MSCs could improve fat retention and promote angiogenesis in fat grafts. Through applying a bioimaging system for BM-MSCs in vivo, performing macrophage depletion experiments, and applying an IL-10 neutralizing antibody to inhibit IL-10 secretion in vitro, we confirmed that the therapeutic effects of BM-MSCs in fat grafting could be due to their induction of macrophage polarization. In addition, the key role of IL-10 in macrophage polarization was determined.

## Methods and materials

### Tissue sources and cell culture

Adipose tissue samples were obtained from healthy donors undergoing liposuction at the Department of Plastic Surgery, First Affiliated Hospital of the Air Force Military Medical University.

RFP-labelled BM-MSCs and BM-MSCs from OriCell strain C57BL/6 mice were purchased from Cyagen Biosciences, Inc. (Guangzhou, China). RAW264.7 mouse monocytes/macrophages were purchased from the Stem Cell Bank, Chinese Academy of Sciences. Both cell types were maintained at 37 °C in a humidified 5% CO_2_ incubator and were cultured in high-glucose DMEM (Gibco) supplemented with 10% FBS (Gibco) and penicillin–streptomycin (100 µg/ml). Cells from passages 3–6 were used for all experiments.

Immunophenotypic characterization of mouse BM-MSCs was performed via flow cytometry. Specifically, BM-MSCs were evaluated by FACS and were showed to be positive for CD29, CD90 and CD44 and negative for the haematopoietic markers CD34 and CD45 (BioLegend, San Diego, CA). The trilineage differentiation potential of BM-MSCs was evaluated by staining according to the manufacturer’s instructions. Morphology was evaluated by Oil Red O staining for adipogenesis, Alizarin Red S staining for osteogenesis and Alcian Blue 8GX staining for chondrogenesis.

### Nude mice fat grafting model

All animal experiments were approved by the Institutional Animal Ethics Committee Laboratory, following the guidelines Center of Air Force Military Medical University (Xi’an, China). Nude mice were purchased from the Experimental Animal Center of Air Force Military Medical University (Xi’an, China) and used to establish a fat grafting model.

In the first set of experiments, 48 nude mice were assessed for the effect of BM-MSCs or PBS on fat grafting. The nude mouse fat graft model was previously described [16]. Briefly, the mice were split into two groups: (1) the PBS group and (2) the BM-MSC group. In the PBS group, a mixture of 0.3 mL (or 0.3 g/spot) of fat particles with 200 µL of PBS was injected into the left side of the back. In the BM-MSC group, a mixture of 0.3 mL (or 0.3 g/spot) of fat with 200 µL of PBS premixed with 1 × 10^6^ BM-MSCs was injected on the other side. As reported previously, both injections were performed in the dorsal region of each mouse at two sites using 14-G needles. After the injections, the skin wounds were sutured with 6–0 nylon, and antibiotic ointment was applied to the wound surface. On days 14, 30 and 90 post-grafting, fat tissues were acquired, and digital photographs were taken with an Olympus digital camera. Additionally, the fat weight was measured with an electronic balance.

In the second set of experiments, 12 nude mice were used to assess the role of macrophages in the BM-MSC-induced improvement in fat retention. To deplete macrophages after fat grafting, the mice were randomly divided into four groups: (1) the PBS + ctrl.Lipo group (2) the PBS + clo.Lipo group (3) the BM-MSCs + ctrl.Lipo group (4) and the BM-MSCs + clo.Lipo group. Clodronate-containing liposomes or control PBS-containing liposomes were subcutaneously injected at 0.5 ml/100 g of body weight into both transferred fat grafts of each mouse every other day for a total of seven times. Fat tissue was then collected from the mice for immunofluorescence staining on days 7 and 14 after macrophage depletion.

### Stem cell tracing

An IVIS bioluminescence imaging system was applied to monitor the fluorescence intensity of BM-MSCs in fat grafts. BM-MSC-RPF mice were placed in the imaging dark box platform after being anaesthetized using an inhaled anaesthesia system, and the system software controlled the lifting of the platform to a suitable field of vision. Imaging was performed in a dark room on days 1, 3, 5, 7 and 14 after injection. Living Image® 4.3.1 software was used to acquire and analyse images.

### Graft retention and H&E examination

Mice were sacrificed at the designated time after grafting. The transferred fat was weighed, and the retention rate was calculated based on weight. That is, the weight at the sampling time was compared to the initial weight. Graft tissues were dissected from each mouse. The fat tissue was fixed in 4% formaldehyde for further histological evaluation by haematoxylin and eosin (H&E) staining following a standard protocol (*n* = 6) [17]. Samples were embedded in paraffin and cut into 5-μm-thick sections.

The integrity of adipose tissue, the presence of cysts and vacuoles and the degree of fibrosis were determined by H&E staining, and samples were imaged and observed using an inverted fluorescence microscope. Each parameter was evaluated with reference to previous literature by a second blinded observer [8].

### Immunofluorescence staining

Immunofluorescence staining of 5-μm formaldehyde-fixed and paraffin-embedded fat tissue sections was applied according to standard procedures. In brief, cells were incubated at 4 °C overnight with the following primary antibodies: anti-CD31 (rabbit IgG, 1:200), anti-MAC-2 (rat IgG, 1:200), anti-F4/80 (rabbit IgG, 1:100), anti-CD206 (rat IgG, 1:400) and anti-RFP (rat IgG, 1:200). Any residual primary antibody was washed away, and the bound primary antibody was reacted with corresponding goat anti-rabbit secondary antibodies. All primary antibodies were purchased from Abcam (Cambridge, UK). Alexa Fluor 448- and Alexa Fluor 594-conjugated goat anti-rabbit and goat anti-rat secondary antibodies were purchased from Invitrogen. Nuclei were visualized with DAPI (5 µg/mL), and images were acquired using a confocal microscope.

### Quantitative real-time polymerase chain reaction (RT-qPCR)

Total RNA was extracted from 50 mg of fat tissue using TRIzol reagent (Invitrogen, USA), and RNA quantity and quality were determined by a Nanodrop. The isolated RNA was reverse transcribed into cDNA using a DNA synthesis kit (TaKaRa, Shiga, Japan). Subsequently, 50 ng of cDNA was used for RT-qPCR with SYBR-green (TaKaRa, Shiga, Japan) and an Applied Real-Time PCR instrument. High-stringency primer pairs for mouse macrophage differentiation markers and mouse angiogenesis-related genes were used. Data were analysed with the 2^−ΔΔCt^ relative quantification technique using GAPDH expression as a normalization control. PCR amplifications were performed using specific primers: for IL-1β, 5′-TCCAGGATGAGGACATGAGCAC (sense) and 5′-GAACGTCACACACCAG-CGTTA(antisense); for VEGF, 5′- ACATTGGCTCACTTCCAGAAACAC(sense) and 5′-TGGTTGGAACCGGCATCTTTA(antisense);for IL-6, 5′-CCACTTCAAGTCGG-AGGCTTA-3′(sense)and 5′-TGACAGTGCATCATCGTTGTTC-3′(antisense); or IL-10,5′-CAAAGGACCAGCTGGACAACA-3′(sense)and 5′-GCAACCCAAGTA-ACCCTTAAAGTC-3′(antisense); for TNF-α, GACCCTCACACTCAGATCATCT-TCT-3′(sense)and 5′-CCACTTGGTGGTTTGCTACGA-3′(antisense); for GAPDH, 5′-ATGGTGAAGGTCGGTGTGA-3′ (sense) and 5′-CTCCACTTTGCCACTGCAA (antisense), All mRNA levels were normalized to that of GAPDH.

### BM-MSC conditioned medium (BM-MSC-CM) collection

BM-MSC-CM was collected from passage 3 BM-MSC cultures. Briefly, BM-MSCs were seeded at a density of 1 × 10^6^ cells in a 100-mm dish with 10% knockout serum in culture medium. After 24 h of incubation, the conditioned medium (CM) was collected by centrifugation for 10 min at 1500 g, and the collected supernatant (CM) was stored at − 80 °C.

### In vitro effects of BM-MSCs on macrophages

The effects of BM-MSCs on macrophages were assessed. In total, 2 × 10^5^ Raw 264.7 cells were transferred to 6-well culture plates with a 0.4-µm pore size (Corning) and cocultured with 2 × 10^5^ BM-MSCs seeded in the upper insert. Moreover, we cultured Raw264.7 cells in mixed medium consisting of 50% high-glucose DMEM and 50% BM-MSC-CM. After 2 days, the supernatant of the macrophages in the bottom insert was collected for immunofluorescence testing, and harvested RNA was used to evaluate macrophage gene expression.

### Enzyme-linked immunosorbent assay (ELISA) assay

The levels of IL-10 within the BM-MSCs-CM were analysed using ELISA kits (R&D Systems, Minneapolis, MN) according to the manufacturer’ instructions, and the total protein was assessed by the BCA method.

### Statistical analysis

Statistical analyses were conducted using GraphPad Prism 8.0, and the mean ± SEM are presented. Student’s t test was used for calculation of statistical differences between two groups, and *p* values less than 0.05 (*p* < 0.05) were defined as statistically significant.

## Results

### Characterization of BM-MSC

**Fig. 1:**
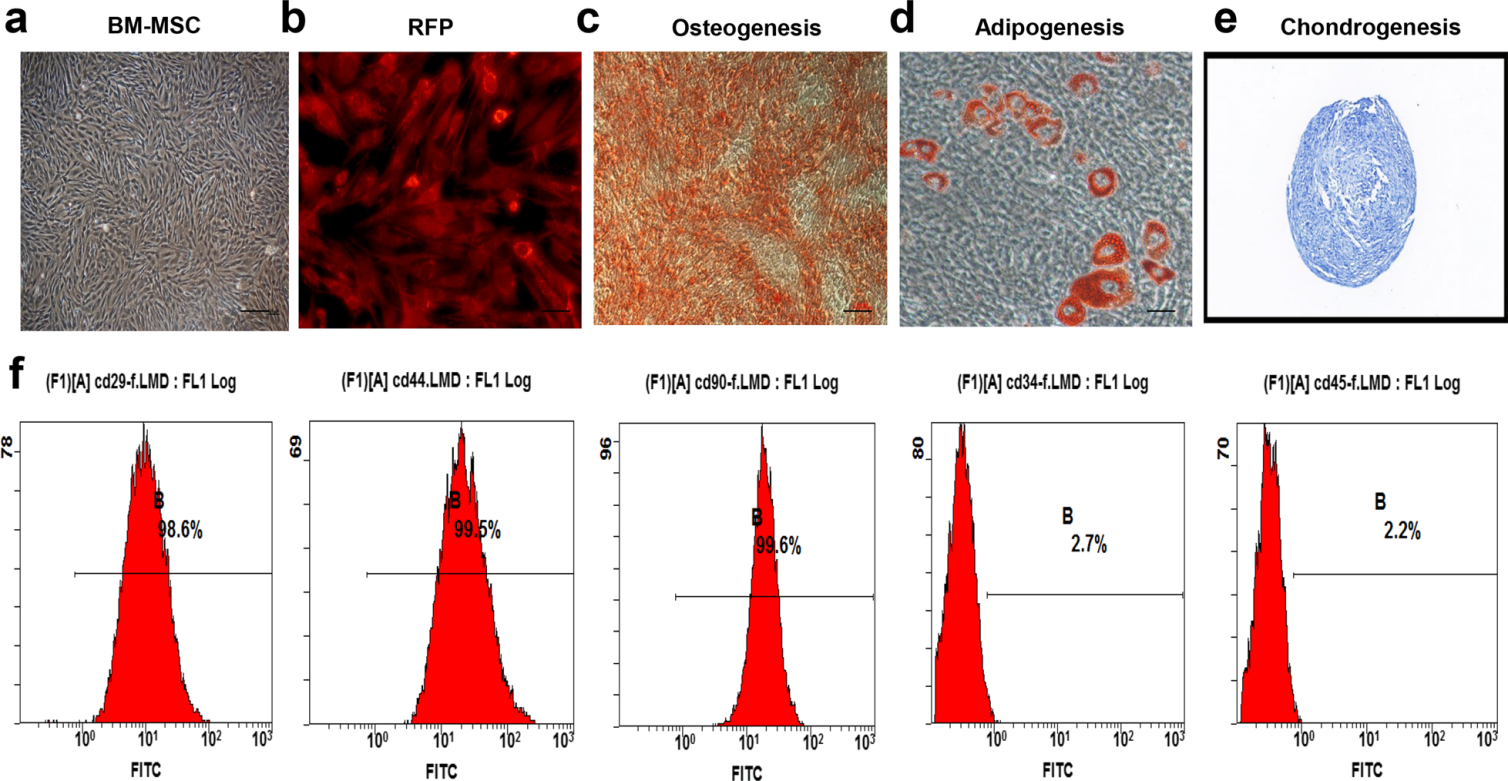
Characterization of BM-MSC. **a**, **b** The morphology of BM-MSCs was observed under a light microscope, and the morphology of BM-MSCs expressing RFP was photographed using a fluorescence microscope. **c**–**e** BM-MSCs were stained with oil red O, alizarin red and Allicin blue, indicating that could differentiate into adipocytes, osteoblasts or chondroblasts, respectively. **f** Flow cytometry analysis showed that these cells were highly enriched for CD29, CD44 and CD90, but negative for CD34 and CD45

BM-MSCs and BM-MSCs-RFP at 80–90% confluence were photographed using an inverted microscope. Most of the cells appeared to have heterogeneous fibroblastic-like appearances (Fig. 1a), and BM-MSCs-RFP showed strong red fluorescence (Fig. 1b). Furthermore, differentiation experiments confirmed that these BM-MSCs could differentiate into adipogenic, osteogenic and chondrogenic lineages (Fig. 1c–e). Isolated BM-MSCs were also used to verify the identity of these using specific markers by flow cytometric, namely, based on the expression of CD29, CD90 and CD44. These were positive in BM-MSC_S_, while CD34 and CD45 were negative (Fig. 1f).

### BM-MSC increase the retention rate of fat graft

**Fig. 2:**
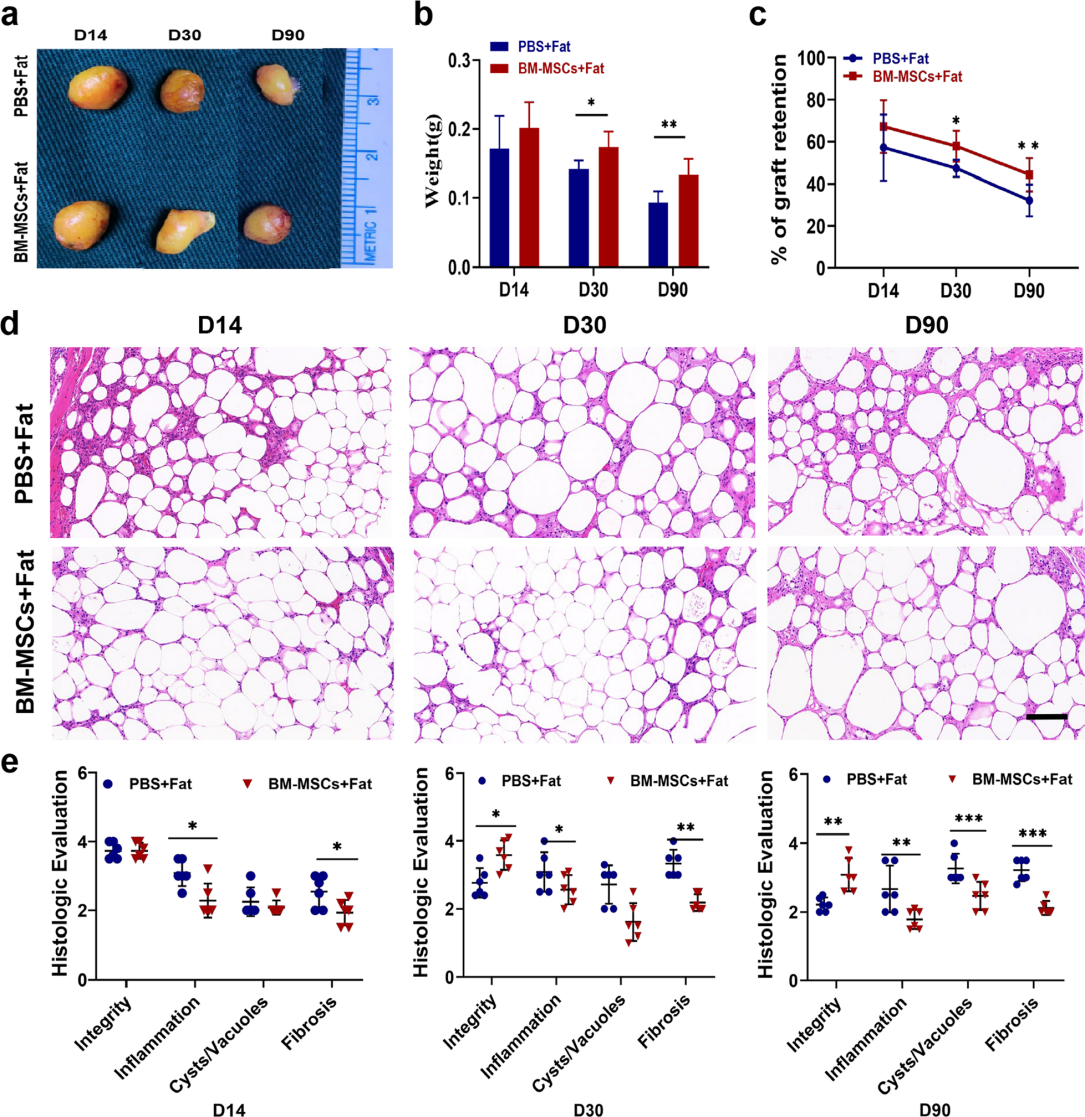
BM-MSCs can improve the retention rate of fat grafting. **a** Representative images from the mixed transplantation of fat grafts with PBS or BM-MSCs. **b, c** Percentage of fat weight and graft weight mixed with PBS or BM-MSCs. **d** Histological images (H&E staining) of fat grafting treated with PBS or BM-MSCs on day 14, 30 or 90. Scale bar 50 um. **e** According to the results from PBS or BM-MSCs and H&E staining after fat transfer, the integrity of fat, cysts, vacuoles and fibrosis were scored histological. *n* = 8 per group. * *P* < 0.05, * * *P* < 0.01* * * *P* < 0.001 and * * * * *P* < 0.0001

We previously reported that BM-MSCs isolated from rabbits had a positive effect on fat grafts. To confirm whether BM-MSCs isolated from mice are as effective as rabbit BM-MSCs in fat grafts, Coleman fat mixed with either PBS or BM-MSCs was transplanted subcutaneously into the left and right sides of nude mice. On day 14, there were no significant differences between the two groups in terms of fat retention (Fig. 2a–c). However, the weight of the transferred fat in the BM-MSC group was significantly greater than that of the transferred fat in the PBS group on day 30 and day 90 (Fig. 2b). The weight retention rate of fat grafts showed a consistent trend (Fig. 2c). These results confirmed that the retention rate of fat grafts in mice was significantly higher for grafts mixed with BM-MSCs than for grafts mixed with PBS from day 30 to day 90.

To further validate the effects of BM-MSCs, H&E staining was performed on days 14, 30, and 90 post transplantation. As shown in Fig. 2d–e, the infiltration of inflammatory cells was observed in both groups. However, the infiltration of inflammatory cells in the PBS group was consistently higher than that in the BM-MSC group. Interestingly, the PBS group showed infiltration of large inflammatory cells on day 14, and severe fibrosis was observed at the later stage of fat grafting compared with the BM-MSC group. In addition, we observed that the BM-MSC group had significantly higher fat integrity scores, fewer cysts, and less fibrosis on day 90 post transplantation (Fig. 2e). These results reveal that BM-MSCs reduce the infiltration of inflammatory cells in the early stage of grafting and lead to better fat quality in the later stage.

### BM-MSCs enhanced angiogenesis in fat grafts and were associated with macrophages

**Fig. 3:**
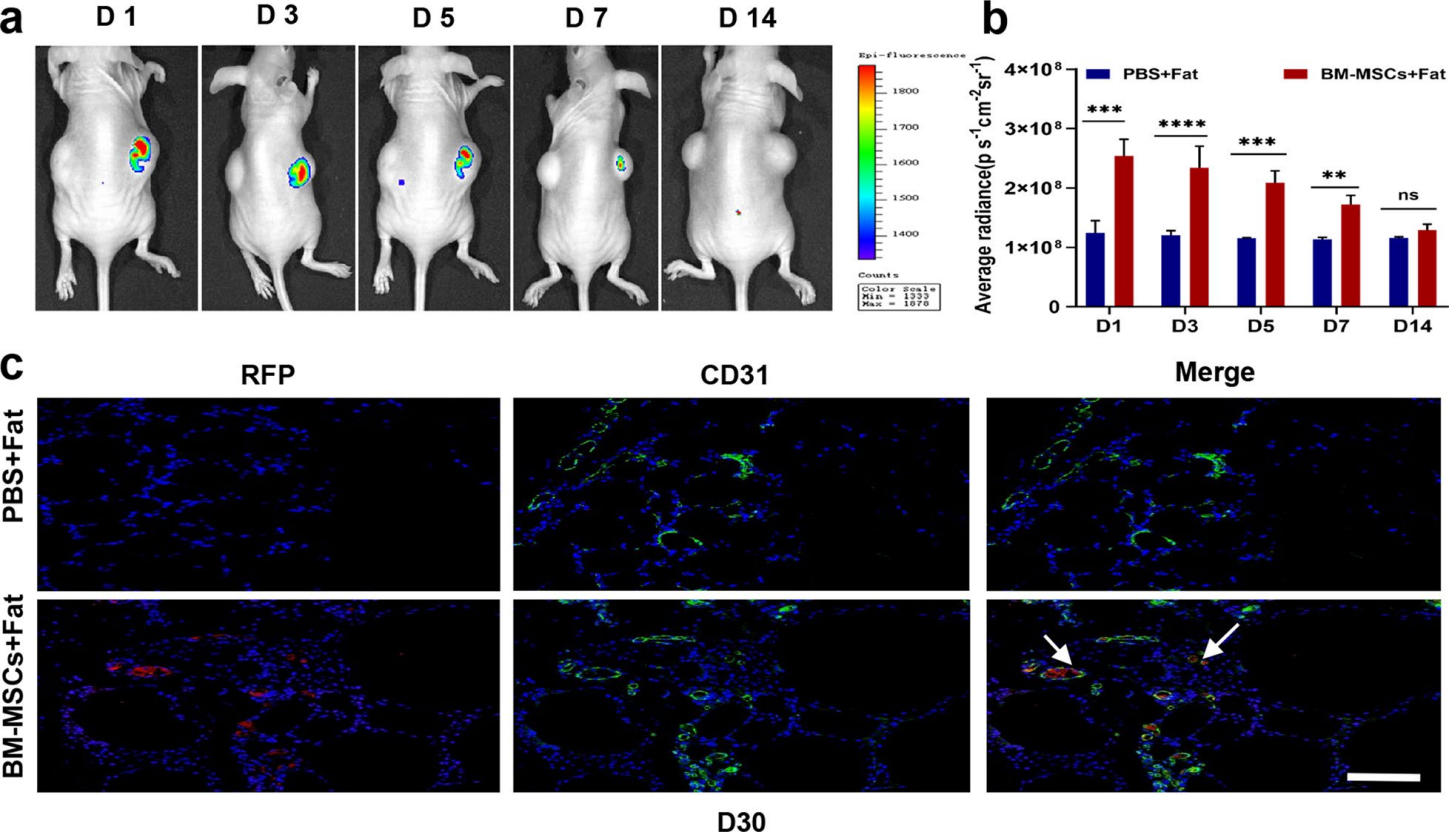
In vivo detection of the distribution of mesenchymal stem cells (BM-MSCs) in fat transplantation. **a** Continuous monitoring of the BM-MSCS-RFP signal after transplantation. **b** The results showed that BM-MSCS-RFP fluorescence was retained for 7 days, and the signal intensity was not detected in the transplanted fat after 7 days. **c** Immunofluorescence staining with RFP antibody and anti-CD31 to label transplanted MSCs and endothelial cells in adipose tissue sections. Scale bar 100 um. *n* = 6 per group. * *P* < 0.05, * * *P* < 0.01* * * *P* < 0.001

**Fig. 4:**
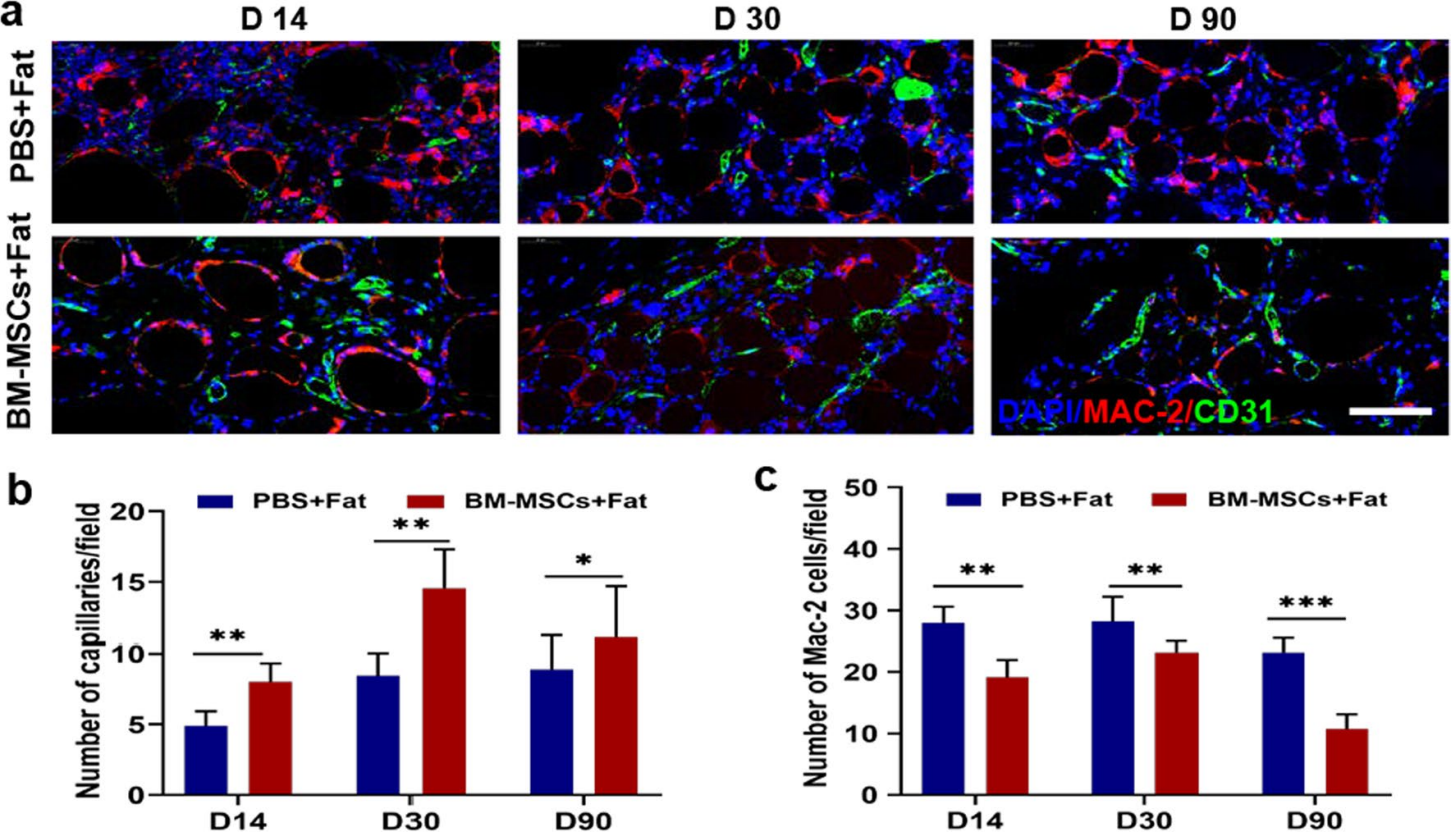
Immunofluorescence analysis of the interaction between blood vessels and macrophages at different time points with PBS or BM-MSCs. **a** The effects of PBS or BM-MSCs on transplanted fat endothelial cells and macrophages were labelled with CD31 (green) and MAC2 (red) staining, respectively. Scale bar 50 um. **b** Compared with the PBS control group, BM-MSCs could promote the vascularization of transplanted fat after transplantation on days 14, 30 and 90. **c** Compared with the PBS control group, BM-MSCs could reduce the number of inflammatory cells. *n* = 6 per group *P < 0.05, **P < 0.01***P < 0.001

To evaluate the pro-angiogenic potential of BM-MSCs in fat grafts, CD31 immunofluorescence staining was used to label vascular endothelial cells in the tissue around each transferred graft. The results of CD31 staining indicated that the number of capillary cells in the fat space in the BM-MSC group was significantly higher than that in the PBS group on days 14, 30 and 90 (Fig. 4a, b). However, we tracked RFP-labelled stem cells in vivo with an IVIS bioluminescence imaging system and found that the number of stem cells in transferred graft, decreased sharply within 1 week (Fig. 3a, b); these cells were almost undetectable after one week, but a small number of CD31 + cells and a low RFP signal were still present at 30 days. Furthermore, immunofluorescence staining showed that most CD31 + cells existed in the Mac2 + region (Fig. 4a, c). These results indicate that BM-MSCs promote angiogenesis not only through the direct transformation of stem cells but also possibly through effects on macrophages.

### Macrophage depletion reduced the effect of BM-MSCs on fat grafting in nude mice

**Fig. 5:**
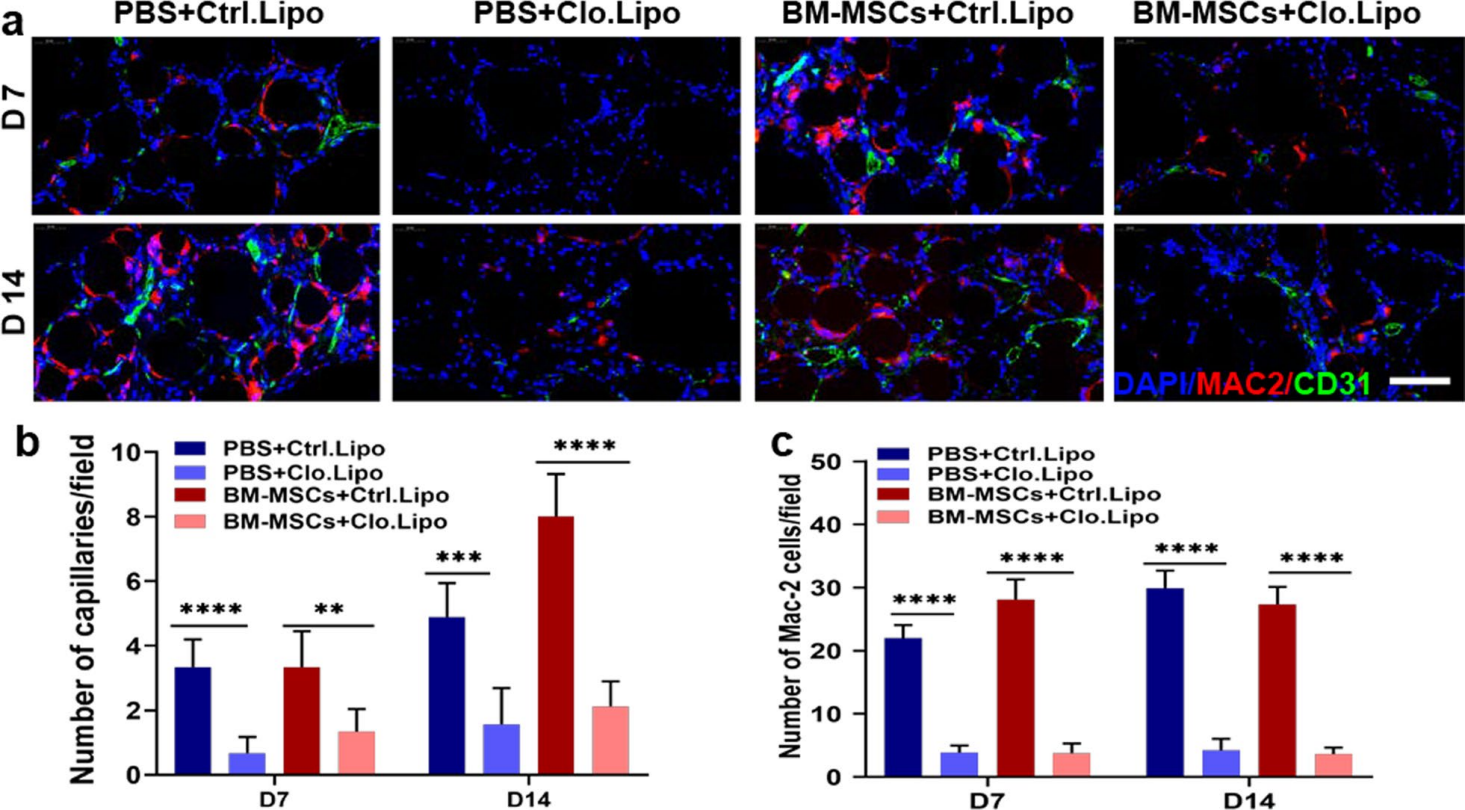
Macrophage depletion reverses the angiogenesis effect of BM-MSCs in fat grafting. **a** 7 days and 14 days after PBS or BM-MSCs treatments, MAC-2 (red) and CD31 (green) were used to stain control or clodronate liposome-treated adipose tissue sections to label endothelial cells and macrophages, respectively. Scale bar 50 um. **b** After clodronate liposome treatment, MAC-2 dropped sharply in the PBS and BM-MSCs groups. **c** Compared with the macrophage depletion group, the blood vessels in the transplanted fat were significantly reduced. *n* = 6 per group. *P < 0.05, **P < 0.01, ***P < 0.001 and ****P < 0.0001

To investigate the pro-angiogenic effects of macrophages on BM-MSCs, clodronate-containing liposomes or PBS-containing liposomes were used to deplete macrophages in vivo. The results showed that clodronate liposomes effectively depleted macrophages in the BM-MSC group. Notably, the clodronate-containing liposome group showed the lowest number of capillaries (Fig. 5a, b). Overall, these results indicated that macrophages were necessary for the promotive effect of BM-MSCs on fat angiogenesis.

### The proangiogenic effect of BM-MSCs in fat grafts is associated with M2 macrophage polarization

**Fig. 6:**
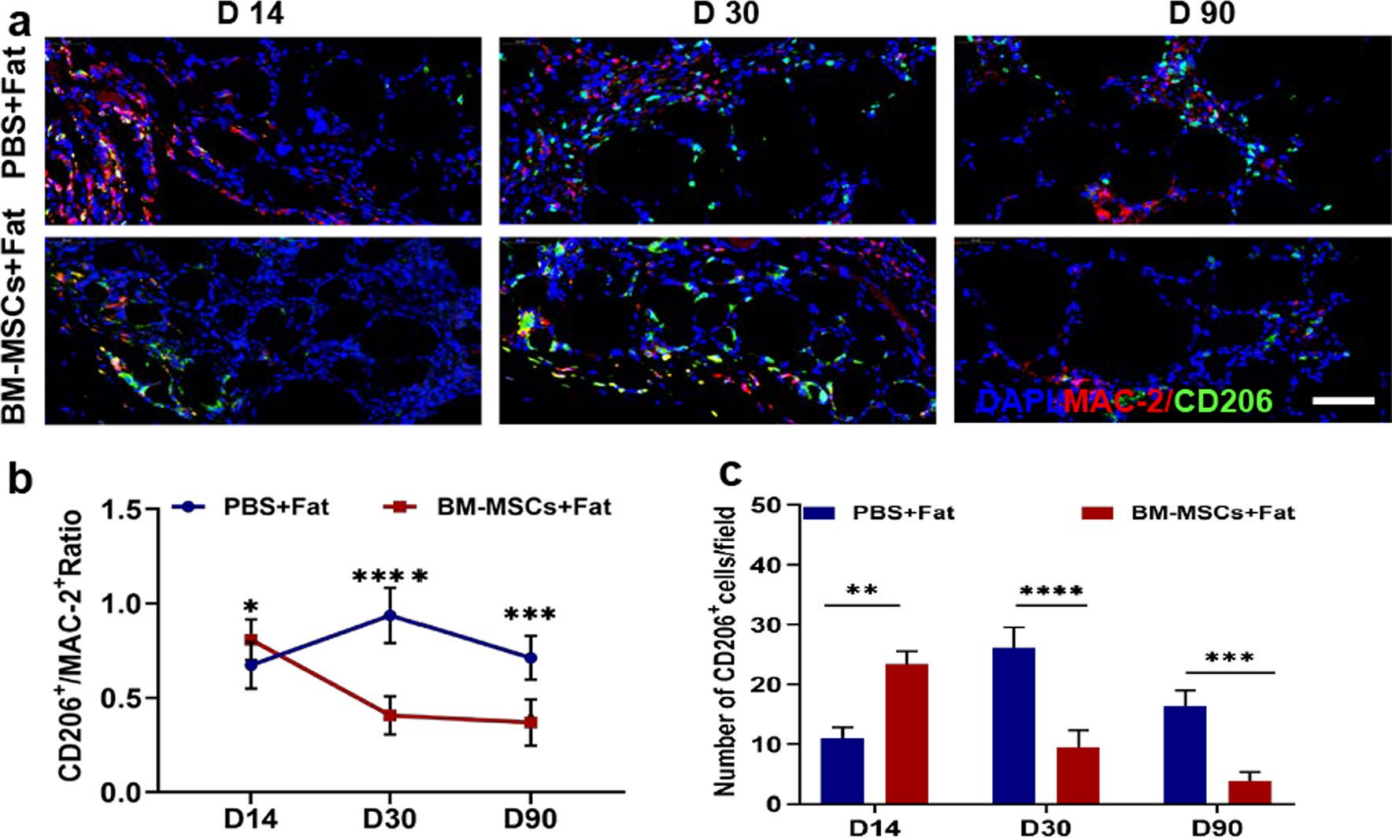
BM-MSCs increased the percentage of M2 macrophages in transplanted fat. **a** The effects of PBS or BM-MSCs on transplanted macrophages and M2 macrophages were labelled with MAC2 + (red) and CD206 + positive (green) staining. Scale bar 50 um. **b**, **c** Compared with the PBS control group, the ratio of CD206 ^+^ M2 macrophages increased rapidly on days 14 of transplantation in the BM-MSCs group, and stabilized on days 30 and 90. The ratio of CD206 + M2 macrophages was still high in PBS group. *n* = 6 per group. *P < 0.05, **P < 0.01, ***P < 0.001 and ****P < 0.0001

Macrophage-mediated inflammation and macrophage polarization state play important roles in fat grafting. Macrophage infiltration into adipose tissues was evaluated using MAC-2 immunostaining. As shown in Fig. 6a, the expression level of MAC-2 in the PBS group was significantly higher than that in the BM-MSC group on day 14.

To evaluate the effect of BM-MSCs on the phenotype of infiltrating macrophages in transplanted fat, immunostaining for MAC-2 and CD206 (M2 macrophage markers) was performed after transplantation. The BM-MSC group contained a significantly higher percentage of MAC-2 + CD206 + macrophages than the PBS group on day 14 post transplantation (Fig. 6b). However, this trend became less apparent over time. Furthermore, the proportion of CD206 + cells was significantly different on day 14 in the early stage after transplantation in the BM-MSC group (Fig. 6c). These results showed that BM-MSCs play a stage-dependent role in macrophage recruitment and mobilization. Specifically, BM-MSCs can increase the percentage of M2 macrophages in the early stage of fat grafting.

### BM-MSCs regulate the local inflammatory microenvironment in fat grafts

**Fig. 7:**
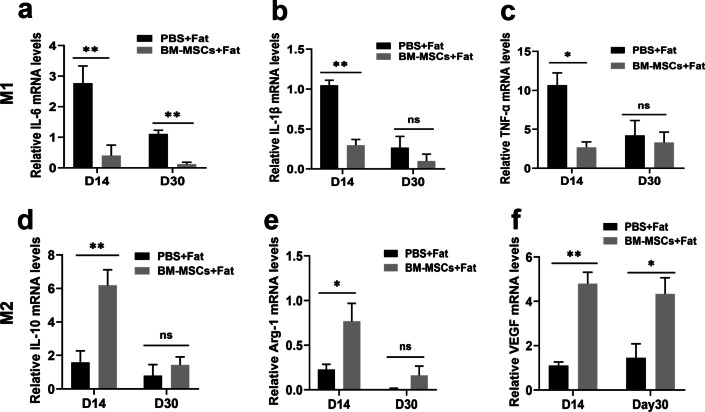
BM-MSCs regulate the inflammatory microenvironment of transplanted fat. The total RNA from transplanted fat treated with PBS and BM-MSCs was extracted, and the expression of inflammatory factors and growth factors in these tissues was quantitatively detected using RT-qPCR. **a** IL-6. **b** IL-1β. **c** TNF-α. **d** IL-10. **e** Arg-1. **f** VEGF. *n* = 6 per group. *P < 0.05 and **P < 0.01

To further clarify the effect of BM-MSCs on the local inflammatory microenvironment during the early stage of fat transplantation, we next evaluated the expression of the M1 markers IL-6, IL-1β and TNF-α; the M2 markers IL-10 and Arg-1; and the vascular growth factor VEGF. The RT–qPCR results indicated that BM-MSCs could significantly decrease the levels of the M1 proinflammatory cytokines TNF-α, IL-1β and IL-6 (Fig. 7a-c) and significantly increase the expression of the anti-inflammatory cytokines IL-10, VEGF, and Arg-1 (Fig. 7d–f). These results suggest that BM-MSCs can influence macrophage polarization and regulate the local microenvironment by increasing the proportion of M2 macrophages.

### BM-MSCs secreted relatively high amounts of IL-10

**Fig. 8:**
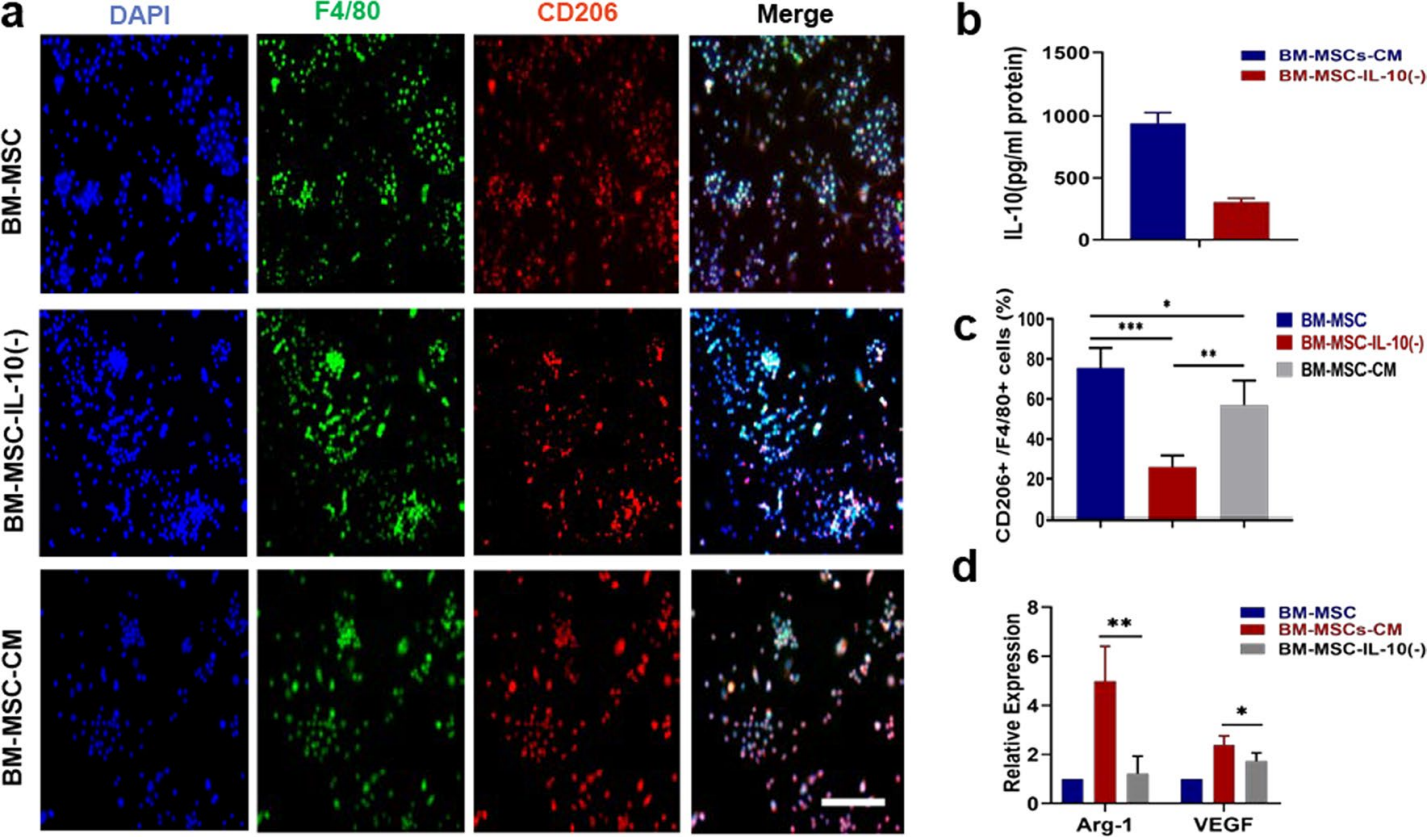
BM-MSCs promoted M2 macrophage polarization via IL-10. **a** Representative immunofluorescence staining for F4/80 and the M2 marker CD206 in BM-MSCs and BM-MSC-CM. Scale bar 100 um. **b** Conditioned medium (CM) from BM-MSCs was collected and analysed for IL-10 by ELISA. **c** Analysis of the percentage of CD206^**+**^ /F4/80^**+**^ on various samples. **d** The mRNA levels of M2 markers in BM-MSCs and BM-MSC-CM. *n* = 6 per group.*P < 0.05, **P < 0.01

In vivo fat grafting in nude mice has revealed the role of macrophages in vascularization of transplanted fat mediated by cytokine release. Moreover, macrophage phenotypic plasticity is related to signals derived from the local microenvironment. According to previous studies, MSCs can affect macrophage polarization through paracrine factors. On this basis, we hypothesized that BM-MSC-CM modulates macrophage polarization through the secreted cytokine IL-10 in BM-MSC-CM. As shown in Fig. 8b, the concentration of IL-10 measured in BM-MSC-CM was over 900 pg/mL. Collectively, these results demonstrate that BM-MSCs can secrete relatively high amounts of IL-10.

### IL-10, as a major component secreted by BM-MSCs, promoted M2 macrophage polarization

To further investigate the role of IL-10 in BM-MSC-induced macrophage polarization, we confirmed that BM-MSC-CM contained a relatively high amount of secreted IL-10. Therefore, we hypothesized that IL-10 is a critical secreted factor of BM-MSCs that alters macrophage phenotype. We neutralized IL-10 in BM-MSC-CM, with double immunocytochemical staining and RT-qPCR were applied to assess macrophage polarization. As shown in Fig. 8a, c, the numbers of CD206 + cells in cultures treated with BM-MSC-CM were increased, and the same effects were observed on the levels of Arg-1 and VEGF. Moreover, neutralizing IL-10 in BM-MSCs showed no effect on macrophage phenotype. Taken together, our results demonstrated that BM-MSCs induced M2 macrophage polarization through IL-10.

## Discussion

In recent years, MSCs have been proposed as a promising treatment option to improve graft retention [1, 8]. In this study, we first found that mouse BM-MSCs increased fat graft retention. Second, live cell tracking and fluorescence double staining experiments were proven that only a small number of stem cells differentiated directly into endothelial cells. Finally, the important relationship between angiogenesis and macrophages in fat transplantation was clarified. Our results showed that BM-MSCs act as trainers of macrophages. They modulate the phenotype of macrophages to M2, Which can establish tissue revascularization more quickly and improve the retention rate of fat grafts.

In this study, we successfully modelled fat grafting in nude mice and used BM-MSCs to facilitate fat grafting. We then monitored the appearance of fat, retained weight of the fat graft, and graft retention rates for statistical analysis. The results showed that the grafts containing BM-MSCs were larger in size and heavier, with higher fat retention rates than grafts mixed with PBS. H&E staining showed an interesting pattern: the BM-MSC group had less inflammatory cell infiltration during the early stage of fat grafting on day 14 than the control group, while the control group had more tissue fibrosis in the later stage.

Stem cells act through multiple mechanisms and improve the efficacy of stem cell therapy [18–20]. In addition to the direct transformative and regenerative abilities of transplanted cells, the regulation of immune cells by stem cells plays a key role in tissue regeneration [21]. Many studies have shown that stem cells recruit immune cells and change the early tissue microenvironment to achieve tissue regeneration [22–24]. All the above findings verified the potential benefits of BM-MSCs in fat grafts. We found that the number of BM-MSCs in transferred fat gradually decreased over time, and only a part of neovascularization was directly differentiated by BM-MSCs. This result was consistent with the findings of previous reports [25, 26]. Thus, BM-MSCs probably increased fat retention by regulating endogenous cells or acting through paracrine mechanisms.

Rapid revascularization in the early stage of transplantation is key to promoting fat retention. According to the early literature and our pre-experimental results, we chose 1 × 10^6^ BM-MSC dose for mixed transplantation. At this dose, the animals survived safely after 3 months transplantation. In addition, we found that the retention rate in the graft changed significantly. Immunohistochemistry showed that the retention of fat grafts in the BM-MSC group was attributed to angiogenesis. This observation was consistent with many previous reports. In this study, we selected only a single effective dose, However, whether there is any causative relationship between stem cell dose and blood vessels must be further studied. Rapid and acute infiltration by macrophages may lead to tissue vascularization and regeneration after fat grafting [17]. Macrophages are involved in mediating the therapeutic effect of BM-MSCs, which has been reported in many disease models [27–29]. To determine whether the effect of BM-MSCs was related to macrophages, we performed double immunofluorescence staining for MAC-2 and CD31, and the results showed that MAC-2 + macrophages colocalized with CD31 + endothelial cells in both groups. In addition, macrophage depletion significantly hindered angiogenesis in fat grafts. Thus, the promotion of fat graft vascularization by BM-MSCs may be related to macrophages.

Macrophages also play essential roles in inflammation and regeneration in transferred fat tissues [30, 31]. which is the key regulator of the local inflammatory environment in the early stage of fat grafting [17]. In different microenvironments, macrophages are polarized into different phenotypes, triggering different functions. M2 macrophages are involved in debris removal, angiogenesis, tissue regeneration and wound healing [32]. Studies have shown that additional supplementation with M2 macrophages increases the retention of transplanted fat by enhancing angiogenesis in fat grafts [17]. M2 macrophages have been shown to play a key role in the early neovascularization process; in vitro experiments showed that the expression of angiogenic growth factors and cytokines in M2 macrophages is higher than that in M1 macrophages, and macrophages secrete a large number of angiogenic factors, such as vascular endothelial growth factor, basic fibroblast growth factor and matrix metalloproteinases [33]. Studies have demonstrated the involvement of PLGF and FGF signalling in M2-induced angiogenesis [34].

Thus, increasing the number or proportion of M2 macrophages in the recipient area is an attractive treatment strategy to increase the retention rate of fat grafts. However, it is difficult to enrich M2 macrophages for clinical application. Therefore, researchers have begun to seek alternative strategies to regulate macrophage polarization.

BM-MSCs have been reported to regulate macrophage phenotype in the context several disorders [35–37]. Recently, using a murine model of diabetic wound healing, Zhang et al. demonstrated that BM-MSCs induced the functional restoration of vascular endothelial cells by polarizing macrophages into an anti-inflammatory phenotype [38]. Cytokines in the inflammatory microenvironment are important in macrophage polarization. In this study, we found that BM-MSCs increased the percentage of M2 macrophages in transferred fat and changed the local environment by inhibiting proinflammatory cytokine expression and increasing anti-inflammatory cytokine levels. These results indicate that BM-MSCs can provide a more favourable environment for M2 macrophages. Moreover, we demonstrated the effect of BM-MSC-CM on RAW264.7 cells in vitro. Additionally, a beneficial component in the BM-MSC secretome was identified. We found that BM-MSCs secreted relatively high amounts of IL-10, which exerted an anti-inflammatory effect and promoted tissue regeneration [39] and also induced M1-to-M2 macrophage repolarization. We found that the induction of M2 macrophages by BM-MSC-CM was attenuated by applying a neutralizing anti-IL-10 antibody, further confirming that IL-10 is necessary for BM-MSC-CM-induced M2 macrophage polarization. However, this study only briefly verified the role of IL-10 in vitro, so we will explore the role of this cytokine in a fat graft model in vivo in the future.

## Conclusions

**Fig. 9:**
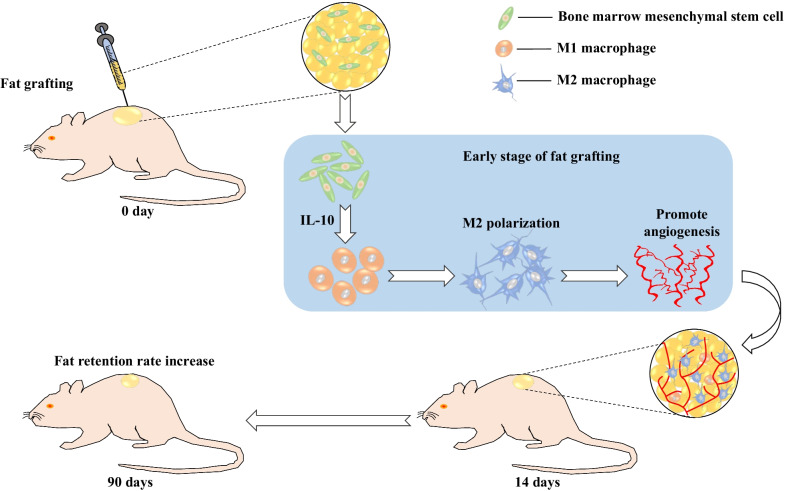
Schematic diagram depicting the potential mechanism by which BM-MSCs can improve the retention rate and enhance angiogenesis in context of fat grafting

In summary, this study showed that BM-MSCs increased the retention rate of fat grafts and enhanced angiogenesis and that these effects may be partly attributed to the repolarization of macrophages into the M2 phenotype via the secretion of IL-10 (Fig. 9). Our results provide novel insight into the role of BM-MSCs in fat grafting..

## Data Availability

All data generated or analyzed during this study are included in this published article**.**

## Abbreviations

BM-MSCs: Bone marrow mesenchymal stem cells
MSCs: Mesenchymal stem cells
Arg1: Arginase 1
H&E: Haematoxylin and eosin
IL6: Interleukin 6
IL-10: Interleukin 10
VEGF: Vascular endothelial growth factor
PBS: Phosphate- buffered saline
LPS: Lipopolysaccharide
IL-1β: Interleukin-1β
TGF-β: Transforming growth factor-β
FBS: Fetal bovine serum
GAPDH: Glyceraldehyde-3-phosphate dehydrogenase
ELISA: Enzyme-linked immunosorbent assay
TNF-α: Tumor necrosis factor-α
RT-qPCR: Quantitative real-time polymerase chain reaction
BM-MSC-CM: Bone marrow mesenchymal stem cells conditioned medium

## References

[1] Borkar R, Wang X, Zheng D, Miao Z, Zhang Z, Li E, Wu Y, Xu RH (2021). Human ESC-derived MSCs enhance fat engraftment by promoting adipocyte reaggregation, secreting CCL2 and mobilizing macrophages. Biomaterials.

[2] Sun JM, Ho CK, Gao Y, Chong CH, Zheng DN, Zhang YF, Yu L (2021). Salvianolic acid-B improves fat graft survival by promoting proliferation and adipogenesis. Stem Cell Res Ther.

[3] Anghelina M, Krishnan P, Moldovan L, Moldovan NI (2006). Monocytes/macrophages cooperate with progenitor cells during neovascularization and tissue repair: conversion of cell columns into fibrovascular bundles. Am J Pathol.

[4] Kato H, Mineda K, Eto H, Doi K, Kuno S, Kinoshita K, Kanayama K, Yoshimura K. Degeneration, regeneration, and cicatrization after fat grafting: dynamic total tissue remodeling during the first 3 months. Plast Reconstr Surg 2014;133(3):303-313. 10.1097/PRS.0000000000000066 24572875

[5] Shao M, Wang D, Zhou Y, Du K, Liu W (2020). Interleukin-10 delivered by mesenchymal stem cells attenuates experimental autoimmune myocarditis. Int Immunopharmacol.

[6] Subhan BS, Kwong J, Kuhn JF, Monas A, Sharma S, Rabbani PS (2021). Amniotic fluid-derived multipotent stromal cells drive diabetic wound healing through modulation of macrophages. J Transl Med.

[7] Zheng Y, Zheng S, Fan X, Li L, Xiao Y, Luo P, Liu Y, Wang L, Cui Z, He F (2018). Amniotic epithelial cells accelerate diabetic wound healing by modulating inflammation and promoting neovascularization. Stem Cells Int.

[8] Zhao J, Yi C, Zheng Y, Li L, Qiu X, Xia W, Su Y, Diao J, Guo S (2013). Enhancement of fat graft survival by bone marrow-derived mesenchymal stem cell therapy. Plast Reconstr Surg.

[9] Wang Z, Chen Y, Zhu S, Chen X, Guan J, Yao Y, Wang X, Li Y, Lu F, Gao J (2020). The effects of macrophage-mediated inflammatory response to the donor site on long-term retention of a fat graft in the recipient site in a mice model. J Cell Physiol.

[10] Eming SA, Murray PJ, Pearce EJ (2021). Metabolic orchestration of the wound healing response. Cell Metab.

[11] Ruytinx P, Proost P, Van Damme J, Struyf S (1930). Chemokine-induced macrophage polarization in inflammatory conditions. Front Immunol.

[12] Guc E, Pollard JW (2021). Redefining macrophage and neutrophil biology in the metastatic cascade. Immunity.

[13] Phipps KD, Gebremeskel S, Gillis J, Hong P, Johnston B, Bezuhly M (2015). Alternatively activated M2 macrophages improve autologous Fat Graft survival in a mouse model through induction of angiogenesis. Plast Reconstr Surg.

[14] Vereb Z, Mazlo A, Szabo A, Poliska S, Kiss A, Litauszky K, Koncz G, Boda Z, Rajnavolgyi E, Bacsi A (2020). Vessel wall-derived mesenchymal stromal cells share similar differentiation potential and immunomodulatory properties with bone marrow-derived stromal cells. Stem Cells Int.

[15] Haideri SS, McKinnon AC, Taylor AH, Kirkwood P, Starkey Lewis PJ, O'Duibhir E, Vernay B, Forbes S, Forrester LM (2017). Injection of embryonic stem cell derived macrophages ameliorates fibrosis in a murine model of liver injury. NPJ Regen Med.

[16] Pedrazza L, Cubillos-Rojas M, de Mesquita FC, Luft C, Cunha AA, Rosa JL, de Oliveira JR (2017). Mesenchymal stem cells decrease lung inflammation during sepsis, acting through inhibition of the MAPK pathway. Stem Cell Res Ther.

[17] Li W, Zhang X, Wu F, Zhou Y, Bao Z, Li H, Zheng P, Zhao S (2019). Gastric cancer-derived mesenchymal stromal cells trigger M2 macrophage polarization that promotes metastasis and EMT in gastric cancer. Cell Death Dis.

[18] Yi H, Wang Y, Yang Z, Xie Z (2020). Efficacy assessment of mesenchymal stem cell transplantation for burn wounds in animals: a systematic review. Stem Cell Res Ther.

[19] Lin H, Sohn J, Shen H, Langhans MT, Tuan RS (2019). Bone marrow mesenchymal stem cells: aging and tissue engineering applications to enhance bone healing. Biomaterials.

[20] Do PT, Wu CC, Chiang YH, Hu CJ, Chen KY (2021). Mesenchymal stem/stromal cell therapy in blood-brain barrier preservation following ischemia: molecular mechanisms and prospects. Int J Mol Sci.

[21] Pittenger MF, Discher DE, Peault BM, Phinney DG, Hare JM, Caplan AI (2019). Mesenchymal stem cell perspective: cell biology to clinical progress. NPJ Regen Med.

[22] Pajarinen J, Lin T, Gibon E, Kohno Y, Maruyama M, Nathan K, Lu L, Yao Z, Goodman SB (2019). Mesenchymal stem cell-macrophage crosstalk and bone healing. Biomaterials.

[23] Zhang S, Chen L, Zhang G, Zhang B (2020). Umbilical cord-matrix stem cells induce the functional restoration of vascular endothelial cells and enhance skin wound healing in diabetic mice via the polarized macrophages. Stem Cell Res Ther.

[24] Guo J, Hu H, Gorecka J, Bai H, He H, Assi R, Isaji T, Wang T, Setia O, Lopes L (2018). Adipose-derived mesenchymal stem cells accelerate diabetic wound healing in a similar fashion as bone marrow-derived cells. Am J Physiol Cell Physiol.

[25] Chow L, Johnson V, Impastato R, Coy J, Strumpf A, Dow S (2020). Antibacterial activity of human mesenchymal stem cells mediated directly by constitutively secreted factors and indirectly by activation of innate immune effector cells. Stem Cells Transl Med.

[26] Wang J, Liu Y, Ding H, Shi X, Ren H (2021). Mesenchymal stem cell-secreted prostaglandin E2 ameliorates acute liver failure via attenuation of cell death and regulation of macrophage polarization. Stem Cell Res Ther.

[27] Mineda K, Kuno S, Kato H, Kinoshita K, Doi K, Hashimoto I, Nakanishi H, Yoshimura K (2014). Chronic inflammation and progressive calcification as a result of fat necrosis: the worst outcome in fat grafting. Plast Reconstr Surg.

[28] Cai J, Feng J, Liu K, Zhou S, Lu F (2018). Early macrophage infiltration improves fat graft survival by inducing angiogenesis and hematopoietic stem cell recruitment. Plast Reconstr Surg.

[29] Liu K, Cai J, Li H, Feng J, Feng C, Lu F (2018). The disturbed function of neutrophils at the early stage of fat grafting impairs long-term fat graft retention. Plast Reconstr Surg.

[30] Cai J, Li B, Liu K, Feng J, Gao K, Lu F (2017). Low-dose G-CSF improves fat graft retention by mobilizing endogenous stem cells and inducing angiogenesis, whereas high-dose G-CSF inhibits adipogenesis with prolonged inflammation and severe fibrosis. Biochem Biophys Res Commun.

[31] Zhang S, Liu Y, Zhang X, Zhu D, Qi X, Cao X, Fang Y, Che Y, Han ZC, He ZX (2018). Prostaglandin E2 hydrogel improves cutaneous wound healing via M2 macrophages polarization. Theranostics.

[32] Li T, He H, Yang Z, Wang J, Zhang Y, He G, Huang J, Song D, Ni J, Zhou X, Zhu J, Ding M (2021). Strontium-doped gelatin scaffolds promote M2 macrophage switch and angiogenesis through modulating the polarization of neutrophils. Biomater Sci.

[33] Yang Y, Guo Z, Chen W, Wang X, Cao M, Han X, Zhang K, Teng B, Cao J, Wu W, Cao P, Huang C, Qiu Z (2021). M2 macrophage-derived exosomes promote angiogenesis and growth of pancreatic ductal adenocarcinoma by targeting E2F2. Mol Ther.

[34] Jetten N, Verbruggen S, Gijbels MJ, Post MJ, De Winther MP, Donners MM (2014). Anti-inflammatory M2, but not pro-inflammatory M1 macrophages promote angiogenesis in vivo. Angiogenesis.

[35] Bai H, Kyu-Cheol N, Wang Z, Cui Y, Liu H, Liu H, Feng Y, Zhao Y, Lin Q, Li Z (2020). Regulation of inflammatory microenvironment using a self-healing hydrogel loaded with BM-MSCs for advanced wound healing in rat diabetic foot ulcers. J Tissue Eng.

[36] Dayan V, Yannarelli G, Billia F, Filomeno P, Wang XH, Davies JE, Keating A (2011). Mesenchymal stromal cells mediate a switch to alternatively activated monocytes/macrophages after acute myocardial infarction. Basic Res Cardiol.

[37] Manole E, Niculite C, Lambrescu IM, Gaina G, Ioghen O, Ceafalan LC, Hinescu ME (2021). Macrophages and stem cells-two to tango for tissue repair?. Biomolecules.

[38] Sok MCP, Baker N, McClain C, Lim HS, Turner T, Hymel L, Ogle M, Olingy C, Palacios JI, Garcia JR (2021). Dual delivery of IL-10 and AT-RvD1 from PEG hydrogels polarize immune cells towards pro-regenerative phenotypes. Biomaterials.

[39] Vannella KM, Wynn TA (2017). Mechanisms of organ injury and repair by macrophages. Annu Rev Physiol.

